# Triple Antithrombotic Therapy after Percutaneous Coronary Intervention (PCI) in Patients with Indication for Oral Anticoagulation: Data from a Single Center Registry

**DOI:** 10.1371/journal.pone.0140101

**Published:** 2015-10-06

**Authors:** Dawid L. Staudacher, Michael Kaiser, Christoph Hehrlein, Christoph Bode, Ingo Ahrens

**Affiliations:** Department of Cardiology and Angiology I, Heart Center University of Freiburg, Freiburg i. Br., Germany; KRH Robert Koch Klinikum Gehrden, GERMANY

## Abstract

Antithrombotic therapy consisting of a dual anti-platelet therapy (DAPT) and oral anti-coagulation (OAC) with a vitamin k antagonist is often referred to as triple therapy. This combined anticoagulation is applied in patients undergoing coronary artery stent implantation while also having an indication for OAC. Triple therapy increases the risk for bleeding events compared to either DAPT or OAC alone and thereby might be associated with adverse outcomes. Clinical data on the frequency of bleeding events in patients on triple therapy from clinical trials derives from pre-selected patients and may differ from the real world patients. We report data on patient characteristics and bleeding incidence of patients dismissed on triple therapy from a single university hospital. Within the time span from January 2000 to December 2012, we identified a total of 213 patients undergoing PCI who were prescribed a triple therapy for at least 4 weeks (representing 0.86% of all patients treated). The usage of triple therapy significantly increased over the observed time period. The average CHA_2_DS_2_-VASc Score was 3.1 ± 1.1 with an average HAS-BLED score of 2.5 ± 0.86 representing a high-risk group for thromboembolic events as well as considerable risk for bleeding events. An on-treatment bleeding incidence of 9.4% was detected, with gastrointestinal and airway bleeding being the most frequent (5.1% and 1.4%, respectively). This is consistent with data from clinical trials and confirms the high risk of bleeding in patients on DAPT plus OAC. 29.0% of all patients receiving triple therapy had an indication for OAC other than non-valvular atrial fibrillation. This substantial patient group is underrepresented by clinical trials and needs further attention.

## Introduction

The coincidence of atrial fibrillation and coronary artery stenosis requiring stent implantation is around 5–7% [1,2]. For these patients, neither oral anticoagulation (OAC) nor dual anti platelet therapy (DAPT) [3] is sufficient by itself to avoid thromboembolism as well as stent thrombosis and re-infarction. The combination of OAC and DAPT, as advocated by current guidelines [4,5], comes at the price of increased bleeding complications [6–8]. Bleeding per se is associated with adverse outcome [9]. A large portion of patients presents with the dilemma of having a high risk of major cardiovascular adverse events (MACE) as well as a high risk of bleeding. Several scoring systems including the HEMORR_2_HAGES, ATRIA, and HAS-BLED scores [10–12] have been introduced in order to quantify bleeding risk and to guide therapy [10]. These scores however have primarily been used to estimate bleeding risk under OAC with vitamin K antagonists and not for patients on triple therapy. Uncertainty still exists concerning bleeding incidence of patients on triple therapy in a real world scenario. We present a 12 year single center experience of patients undergoing PCI while simultaneously requiring OAC who were treated at the Heart Center, University of Freiburg.

## Methods

We retrospectively analyzed all patients treated at our center between 2000 and 2012. Patients were treated at the Heart Center University of Freiburg according to current guidelines. Triple therapy consisting of Aspirin, Clopidogrel and an oral vitamin K antagonist was considered the best treatment option by the physicians for each individual patient.

The work was approved by the Ethics Committee of the University of Freiburg (614/14). Specifically, data analysis was performed and reported anonymously therefore not requiring an informed consent. Before contacting patients for the telephone follow-up, a written informed consensus was provided by mail. The informed consent was returned by patients using a provided postpaid envelope.

**Fig 1:**
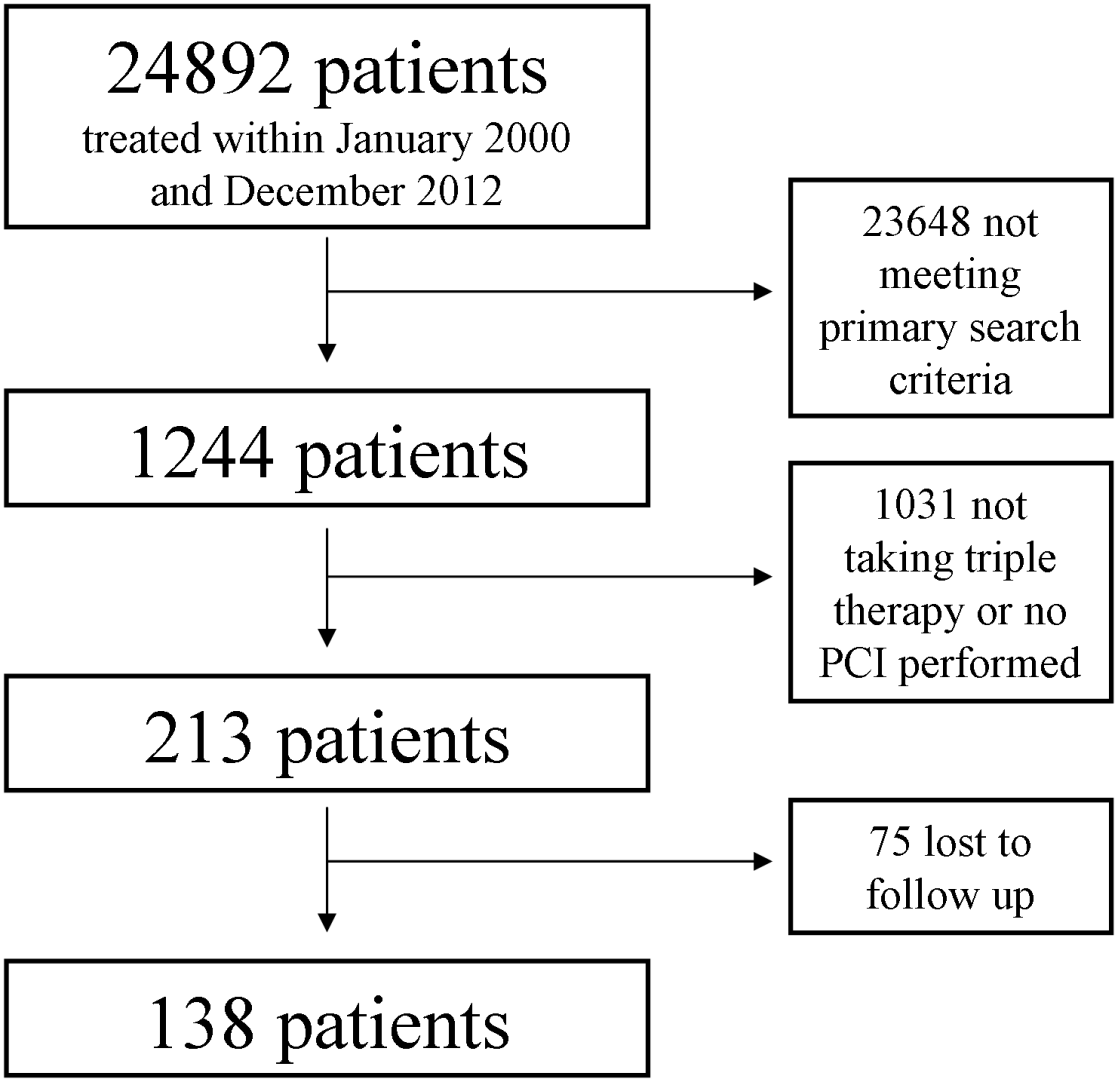
Screening algorithm of patients on triple therapy after stent implantation. A total of 24892 patients were treated within 2000 and 2012. A computerized search returned 1244 hits of which 213 were actually discharged on triple therapy. Complete data is available on 138 patients.

**Fig 2:**
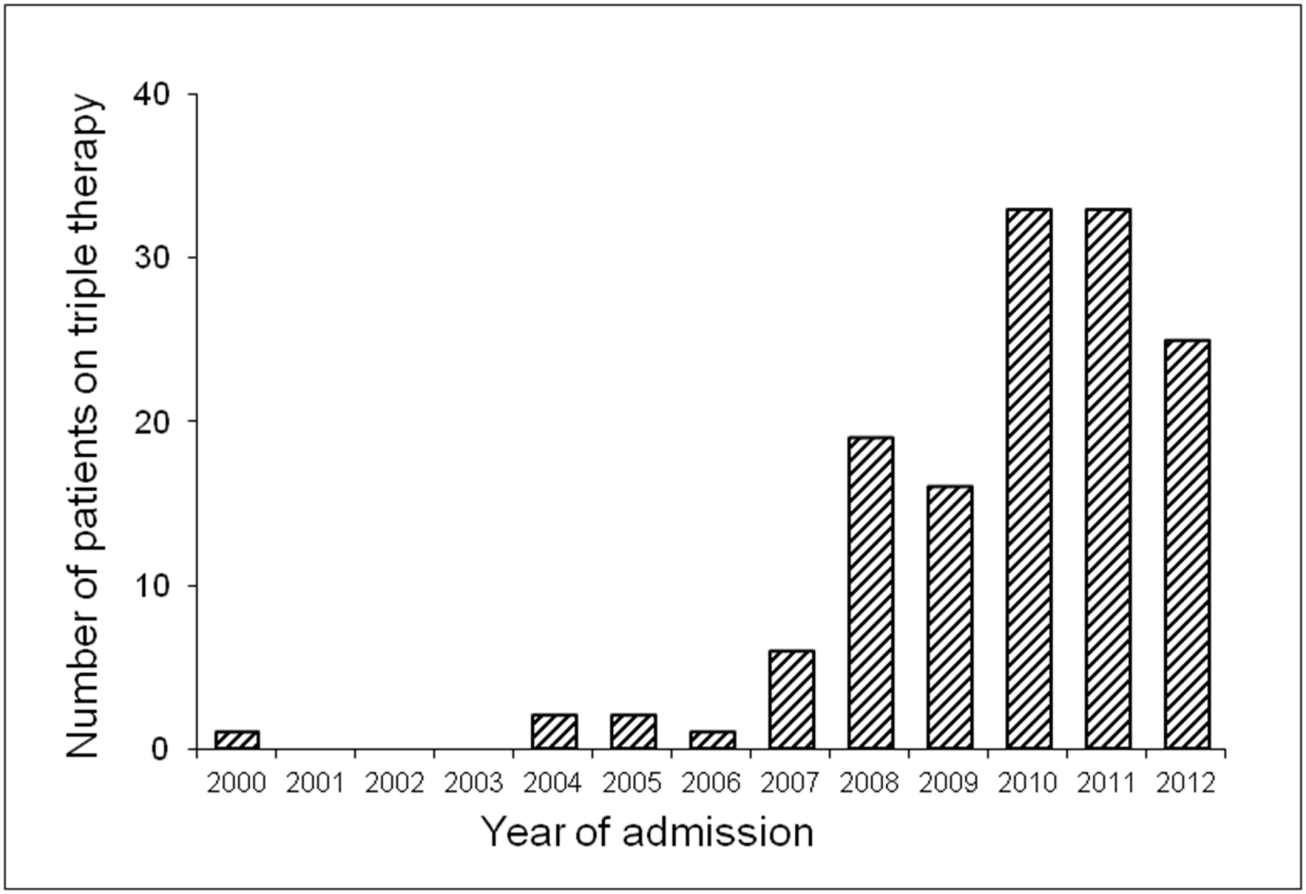
Number of patients discharged on triple therapy by year.

**Fig 3:**
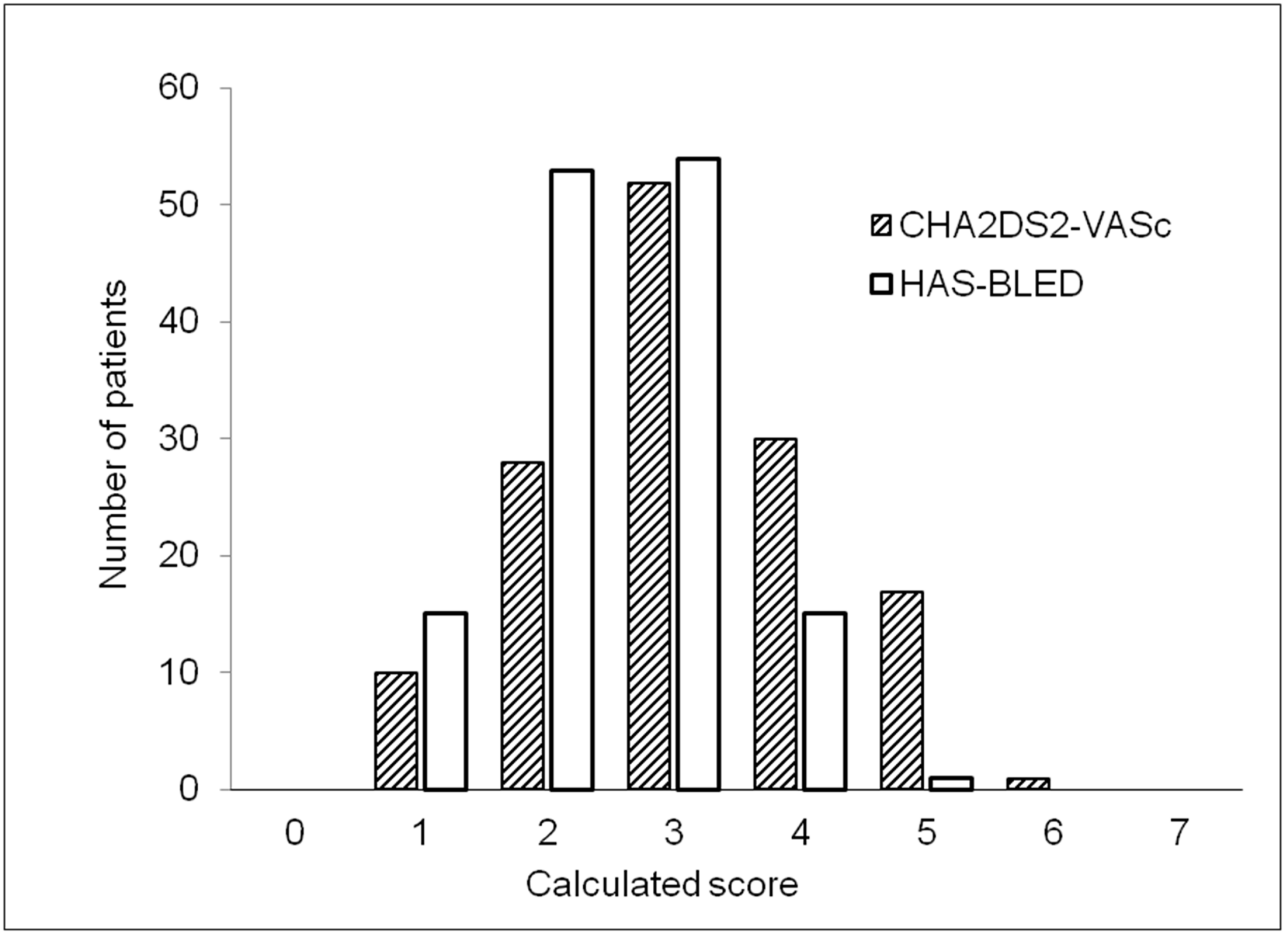
Distribution of the CHA_2_DS_2_-VASc and the HAS-BLED Score of all patients on triple therapy.

Within this time period a total of 24892 individual patients (excluding re-admissions) were treated at the present Department of Cardiology and Angiology I, Heart Center, University of Freiburg, (former: Department of Cardiology and Angiology, University of Freiburg). Using a computerized search, we identified a total of 1244 patients whose medical reports included all search criteria. Search criteria were ‘PTCA and stent implantation’ and ‘phenprocoumon or Marcumar^®^’ and ‘ASS or acetylsalicylic acid or Aspirin^®^’ and ‘clopidogrel or Plavix^®^/Iscover^®^ or ‘ticlopidin or Tiklyd^®^. Through manual review, we identified a total of 213 patients actually receiving triple therapy after PCI. We were able to retrieve follow-up data of a total of 138 patients using medical records and a telephone survey. Only data from patients with complete follow-up are reported (Figs 1 and 2). Data base was based on a tabular listing of patient characteristics using (Microsoft Excel). For some items (like bleeding events), multiple selections were possible while most other items only single selection was possible (like gender). Calculated data included glomerular filtration rate (calculated from the MDRD-formula) and the HAS-BLED [11,12] and CHA_2_DS_2_-VASc [13] Scores. Distribution of the scores is given in Fig 3.

### Definition of bleeding events

**Fig 4:**
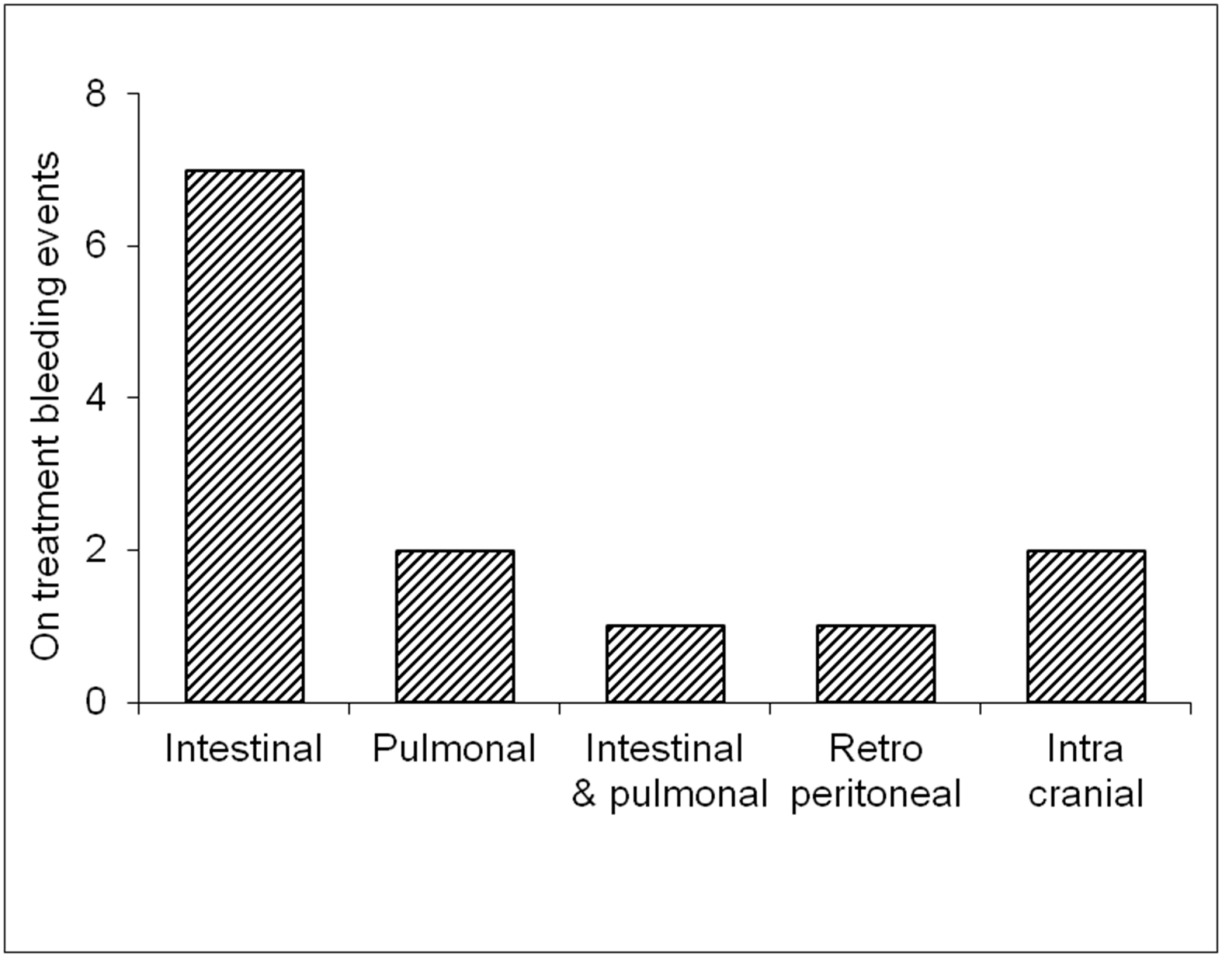
Distribution of bleeding events of patients on triple therapy by bleeding site.

Bleeding events were screened in the medical records from hospital stays at the Heart Center University of Freiburg as well as from a telephone survey. The aim was to detect any clinically significant bleeding event (Fig 4). Specifically, any bleeding being mentioned in the medical records or being remembered by the patient was considered to be a significant bleeding event and therefore recorded. Major bleeds were defined as any bleeding requiring transfusion or medical attention including hospital admission. Only bleeding events on triple therapy were recorded.

### Telephone survey

Long term bleeding events were detected by a telephone survey (ethics approval 614/14). Patients were contacted by telephone and bleeding events were detected using a standardized questionnaire. Specifically, items were: greeting; verification of patient identity; confirmation of agreement to written consensus; adherence to triple therapy; anamnesis of bleeding history; anamnesis of complications like cerebral or myocardial infarction; formal thanks; farewell. The time of the phone call was intended to be as close to the end of the triple therapy treatment as possible in order to detect all bleeding events. Since a time period of 10 years was covered, the average latency between discharge and telephone call was 3.2 years.

## Results

We identified a total of 213 patients discharged on triple therapy between January 2000 and December 2012. Triple therapy consisted of OAC with the vitamin K antagonist phenprocoumon and DAPT with aspirin (100 mg/day) and clopidogrel (75 mg/d). New oral anticoagulants and third generation P2Y_12_ receptor antagonists (prasugrel, ticagrelor) were excluded in the search strategy. Patients on triple therapy represent 0.86% of all individuals undergoing PCI and being discharged during the registered period. We report complete data of 138 patients. Follow up was incomplete for 75 patients, which were excluded from the final analysis (Fig 1). Our patients were predominantly male (79.0%) and had an average age of 73.1 ± 9.8 years. 29 patients (21.0%) presented with a troponin T positive (troponin T > 0.1 ng/ml) ACS as the index event. Patient characteristics are given in Table 1.

**Table 1:** Patient characteristics.

No of patients	138 (100%)
Age	73.1 ± 9.8 years
Male gender	109 (79.0%)
Coronary artery disease	130 (94.2%)
1 vessel disease	27 (19.6%)
2 vessel disease	52 (36.2%)
3 vessel disease	53 (38.4%)
Any stents implanted	127 (92.0%)
bare metal stent	75 (52.2%)
drug eluting stent	55 (39.9%)
drug eluting balloon	7 (5.1%)
Triple Therapy 4 Weeks	65 (47.1%)
bare metal stent	54 (83.1%)
drug eluting stent	8 (12.3%)
drug eluting balloon	3 (4.6%)
Triple Therapy > 4 Weeks	73 (52.9%)
bare metal stent	18 (24.7%)
drug eluting stent	47 (64.4%)
drug eluting balloon	4 (5.5%)
Arial fibrillation	98 (71.0%)
intermittent	29 (21.0%)
chronic	69 (50.0%)
Serum Creatinine	1.28 ± 0.67 mg/dl
eGFR [MDRD]	
> 60	77 (55.8%)
0–60	52 (37.7%)
< 30	7 (5.1%)
ACS	29 (21.0%)
Stable angina	109 (79.0%)

Numbers given represent the absolute number of patients with specific characteristic with percentage given in brackets. Some characteristics like age and hemoglobin are given as mean ± standard deviation.

Concerning indication for OAC, 71.0% of all patients had either chronic or intermittent atrial fibrillation. Other indications for OAC were intra-cardial thrombi after myocardial infarction, recurrent pulmonary embolism, coagulation abnormalities such as factor V mutation and recurrent deep vein thrombosis (compare S1 Table). The average CHA_2_DS_2_-VASc score in our patients was 3.1 ± 1.1 with an average HAS-BLED score of 2.5 ± 0.86 representing a high-risk group for thromboembolic as well as bleeding events. Distribution of both scores is given in Fig 3.

All analyzed patients underwent a coronary angiography within the index hospital stay (Table 1). Stents were implanted in 92.0% of all patients, specifically bare metal stents were implanted in 52.2% and at least one drug eluting stent was used in 39.9%. A drug eluting balloon PCI was performed in 7 patients (5.1%). The duration of triple therapy was restricted to 4 weeks in 47.1% of all patients while being up to one year in 52.9%. In the short therapy group, predominantly BMS (83.1%) were employed while in the long triple therapy group, 64.4% DES were implanted.

A total of 13 patients reported at least one bleeding event within the follow up period representing a bleeding incidence on triple therapy treatment of 9.4%. The most common bleedings were of gastrointestinal and pulmonal origin, representing 76.9% of all bleedings. Two fatal intracranial bleeds have to be reported (incidence 1.45%). Bleeding incidence was 9.2% in patients with and 10.0% in patients without atrial fibrillation respectively (Fig 4).

**Fig 5:**
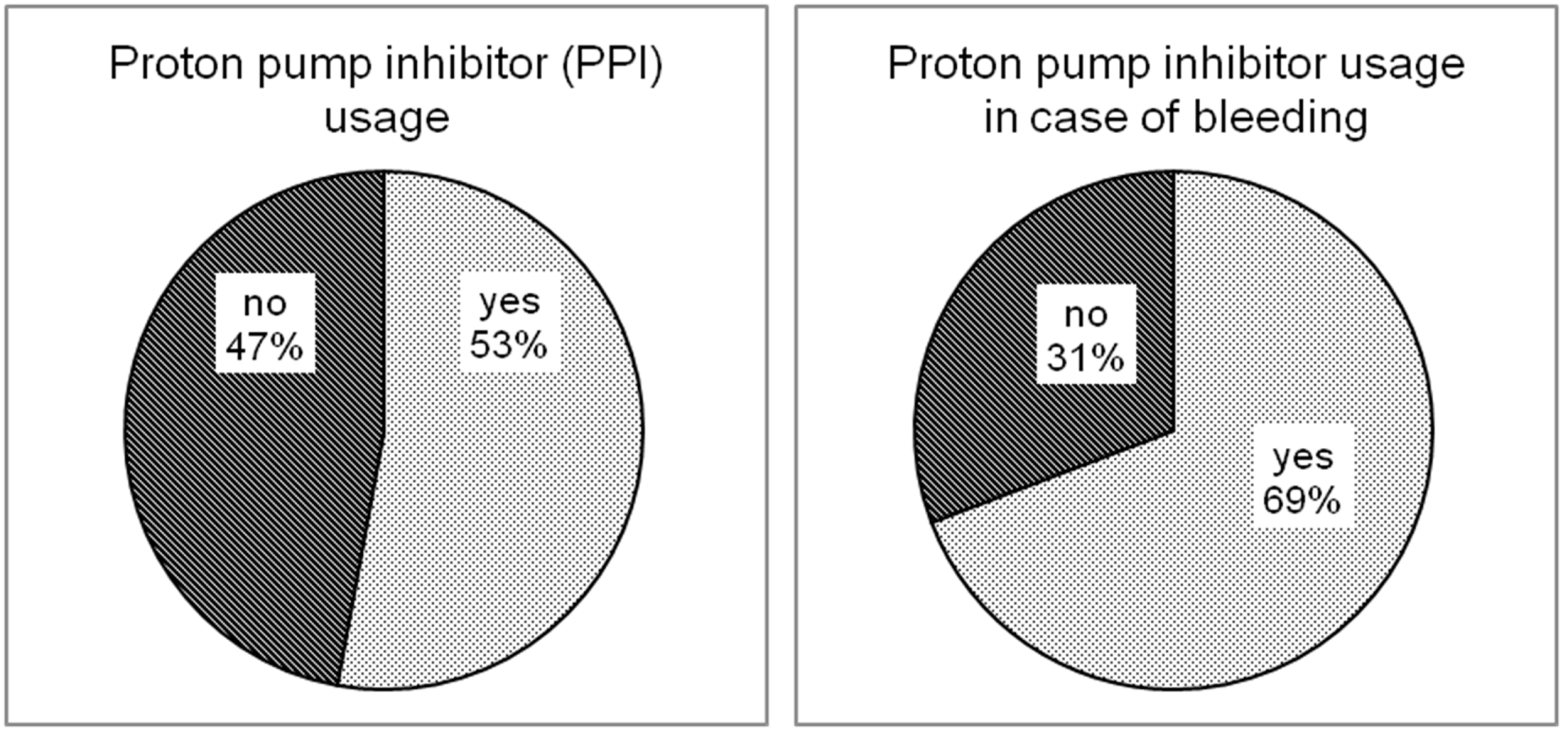
Distribution of proton pump inhibitor usage of all patients and of patients with bleeding events. PPI: proton pump inhibitor.

Since gastrointestinal bleedings were predominant, proton pump inhibitor usage among the analyzed patients was also evaluated (Fig 5). Specifically, 52.9% of all patients dismissed were prescribed a proton pump inhibitor. When considering patients actually reporting any bleeding, we found that proton pump prescription was even higher (69.2%) than in the whole collective.

## Discussion

From January 2000 to December 2012 only 0.86% of all patients dismissed from the Heart Center University of Freiburg were on triple therapy with a recommendation for duration of treatment for at least 4 weeks. Considering the coincidence of atrial fibrillation and CAD in literature is 5–7% [1,2], the real world usage of triple therapy was relatively low. We can demonstrate however a marked increase in triple anti-thrombotic therapy usage over the last years.

Indication for oral anticoagulation was different to non-valvular atrial fibrillation in 29.0% of all patients. This group of patients represents a heterogeneous population largely underrepresented in published clinical trials investigating triple therapy in the setting of PCI. Whether this group of patients has the same risk / benefit ratio concerning triple therapy as atrial fibrillation patients can methodically not be answered with this registry. Bleedings however seem to be comparable in patients receiving triple therapy for atrial fibrillation and those heaving another indication (9.2% and 10.0% respectively).

Applying the HAS-BLED score (which has not been specifically validated in the setting of triple therapy) to our patient collective, the estimated bleeding incidence would be in the range of 4.1%—5.8% [11,12] per year according the average HAS-BLED score of 2.5 in our patients. The detected bleeding incidence while on triple anti thrombotic therapy in our patients however was 9.4%. This is in line with data from the WOEST trial [14], which reported a 12.3% incidence rate of GUSTO moderate to severe bleeding in the triple therapy group. Similar bleeding rates were found in registries, with an incidence of moderate to severe bleeding of 12% to 15% per year [2,6,8]. This highlights again that the HAS-BLED score [11,12] underestimates bleeding incidence in triple therapy patients.

## Study Limitations

This study represents a register analysis of a small subgroup of patients. The patients receiving triple therapy at our institution are highly selected by the physicians. Since our follow up derives from a telephone survey and medical records of our institution only, we most probably underreport bleeding complications. A total of 75 patients have been lost to follow up, which might influence the reported findings. Last, triple therapy always consisted of Aspirin and a second generation P2Y_12_ antagonist (Clopidogrel) and vitamin K antagonist.

## Conclusion

Triple therapy prescription is increasing over the last decade in a real world scenario. Bleeding complications are frequent even under PPI usage. 29.0% of all patients receiving triple therapy have an indication for OAC other than non-valvular atrial fibrillation. This group is under represented by clinical trials. We will need larger trials to evaluate the net benefit for this specific group of patients.

## Supporting Information

S1 Table Indication for the addition of an oral anticoagulant to DAPT after coronary stent implantation other than non-valvular atrial fibrillation. A total of 40 Patients (representing 29% of all patients) received a triple therapy consisting of Aspirin plus Clopidogrel plus a Coumarin for an indication other than non-valvular atrial fibrillation.
(DOCX)

## References

[1] Rubboli A, Bolognese L, Di Pasquale G, Galvani M, La Vecchia L, Maggioni AP (2009) A prospective multicentre observational study on the management of patients on oral anticoagulation undergoing coronary artery stenting: rationale and design of the ongoing warfarin and coronary stenting (WAR-STENT) registry. J Cardiovasc Med (Hagerstown) 10: 200–203. 19377385 10.2459/JCM.0b013e3283212f07

[2] Rossini R, Musumeci G, Lettieri C, Molfese M, Mihalcsik L, Mantovani P, et al (2008) Long-term outcomes in patients undergoing coronary stenting on dual oral antiplatelet treatment requiring oral anticoagulant therapy. Am J Cardiol 102: 1618–1623. 10.1016/j.amjcard.2008.08.021 19064015

[3] Connolly S, Pogue J, Hart R, Pfeffer M, Hohnloser S, Chrolavicius S, et al (2006) Clopidogrel plus aspirin versus oral anticoagulation for atrial fibrillation in the Atrial fibrillation Clopidogrel Trial with Irbesartan for prevention of Vascular Events (ACTIVE W): a randomised controlled trial. Lancet 367: 1903–1912. 16765759 10.1016/S0140-6736(06)68845-4

[4] Hamm CW, Bassand JP, Agewall S, Bax J, Boersma E, Bueno H, et al (2011) ESC Guidelines for the management of acute coronary syndromes in patients presenting without persistent ST-segment elevation: The Task Force for the management of acute coronary syndromes (ACS) in patients presenting without persistent ST-segment elevation of the European Society of Cardiology (ESC). Eur Heart J 32: 2999–3054. 10.1093/eurheartj/ehr236 21873419

[5] O’Gara PT, Kushner FG, Ascheim DD, Casey DE, Chung MK, de Lemos JA, et al (2013) 2013 ACCF/AHA Guideline for the Management of ST-Elevation Myocardial Infarction: Executive Summary: A Report of the American College of Cardiology Foundation/American Heart Association Task Force on Practice Guidelines. Circulation 127: 529–555. 10.1161/CIR.0b013e3182742c84 23247303

[6] Hansen ML, Sorensen R, Clausen MT, Fog-Petersen ML, Raunso J, Gadsboll N, et al (2010) Risk of bleeding with single, dual, or triple therapy with warfarin, aspirin, and clopidogrel in patients with atrial fibrillation. Arch Intern Med 170: 1433–1441. 10.1001/archinternmed.2010.271 20837828

[7] Abraham NS, Hartman C, Richardson P, Castillo D, Street RL Jr., Naik AD (2013) Risk of lower and upper gastrointestinal bleeding, transfusions, and hospitalizations with complex antithrombotic therapy in elderly patients. Circulation 128: 1869–1877. 10.1161/CIRCULATIONAHA.113.004747 24025594

[8] Sorensen R, Hansen ML, Abildstrom SZ, Hvelplund A, Andersson C, Jorgensen C, et al (2009) Risk of bleeding in patients with acute myocardial infarction treated with different combinations of aspirin, clopidogrel, and vitamin K antagonists in Denmark: a retrospective analysis of nationwide registry data. Lancet 374: 1967–1974. 10.1016/S0140-6736(09)61751-7 20006130

[9] Subherwal S, Bach RG, Chen AY, Gage BF, Rao SV, Newby LK, et al (2009) Baseline risk of major bleeding in non-ST-segment-elevation myocardial infarction: the CRUSADE (Can Rapid risk stratification of Unstable angina patients Suppress ADverse outcomes with Early implementation of the ACC/AHA Guidelines) Bleeding Score. Circulation 119: 1873–1882. 10.1161/CIRCULATIONAHA.108.828541 19332461 PMC3767035

[10] Apostolakis S, Lane DA, Guo Y, Buller H, Lip GY (2012) Performance of the HEMORR(2)HAGES, ATRIA, and HAS-BLED bleeding risk-prediction scores in patients with atrial fibrillation undergoing anticoagulation: the AMADEUS (evaluating the use of SR34006 compared to warfarin or acenocoumarol in patients with atrial fibrillation) study. J Am Coll Cardiol 60: 861–867. 10.1016/j.jacc.2012.06.019 22858389

[11] Lip GY, Frison L, Halperin JL, Lane DA (2011) Comparative validation of a novel risk score for predicting bleeding risk in anticoagulated patients with atrial fibrillation: the HAS-BLED (Hypertension, Abnormal Renal/Liver Function, Stroke, Bleeding History or Predisposition, Labile INR, Elderly, Drugs/Alcohol Concomitantly) score. J Am Coll Cardiol 57: 173–180. 10.1016/j.jacc.2010.09.024 21111555

[12] Pisters R, Lane DA, Nieuwlaat R, de Vos CB, Crijns HJ, Lip GY (2010) A novel user-friendly score (HAS-BLED) to assess 1-year risk of major bleeding in patients with atrial fibrillation: the Euro Heart Survey. Chest 138: 1093–1100. 10.1378/chest.10-0134 20299623

[13] Lip GY, Nieuwlaat R, Pisters R, Lane DA, Crijns HJ (2010) Refining clinical risk stratification for predicting stroke and thromboembolism in atrial fibrillation using a novel risk factor-based approach: the euro heart survey on atrial fibrillation. Chest 137: 263–272. 10.1378/chest.09-1584 19762550

[14] Dewilde WJ, Oirbans T, Verheugt FW, Kelder JC, De Smet BJ, Herrman JP, et al (2013) Use of clopidogrel with or without aspirin in patients taking oral anticoagulant therapy and undergoing percutaneous coronary intervention: an open-label, randomised, controlled trial. Lancet 381: 1107–1115. 10.1016/S0140-6736(12)62177-1 23415013

