# Supplementary material for: Triple Antithrombotic Therapy after Percutaneous Coronary Intervention (PCI) in Patients with Indication for Oral Anticoagulation: Data from a Single Center Registry
Source: PLoS One. 2015 Oct 6;10(10):e0140101. doi: 10.1371/journal.pone.0140101 (PMC4595133; doi:10.1371/journal.pone.0140101)
Supplement: S1 Table — A total of 40 Patients (representing 29% of all patients) received a triple therapy consisting of Aspirin plus Clopidogrel plus a Coumarin for an indication other than non-valvular atrial fibrillation. (DOCX) [file pone.0140101.s001.docx]

| Indication | Number of patients |
| --- | --- |
| Apex aneurysm | 4 (10%) |
| Atrial slow flow | 1 (2.5%) |
| Apoplexy | 4 (10%) |
| Artial tachycardia | 1 (2.5%) |
| Aortic valve replacement | 2 (5%) |
| Atrial flutter | 3 (7.5%) |
| Aortic aneurysm | 2 (5%) |
| Decreased LV function | 8 (20%) |
| Factor V mutation | 3 (7.5%) |
| Factor XI and FXII deficiency | 1 (2.5%) |
| Peripheral artery disease | 2 (5%) |
| Paradox embolism (PFO) | 1 (2.5%) |
| Pulmonary embolism | 4 (10%) |
| Valvular atrial fibrillation | 2 (5%) |
| Venous thrombosis | 2 (5%) |

**supplemental table 1: Indication for the addition of an oral anticoagulant to DAPT after coronary stent implantation other than non-valvular atrial fibrillation.** A total of 40 Patients (representing 29 % of all patients) received a triple therapy consisting of Aspirin plus Clopidogrel plus a Coumarin for an indication other than non-valvular atrial fibrillation.
